# Surgical Anatomy

**Published:** 1850-02

**Authors:** 


ART. III.—*Surgical Anatomy.* By JOSEPH MACLISE, Surgeon: with colored plates. Philadelphia: Lea & Blanchard, 1850. In four parts. Price, $2 each part.

We have just received the first part of this splendid work, containing sixteen of the most accurately engraved and beautifully colored plates we have seen in an American book. Originality, except in the mode and style of display, the anatomist of the present day does not attempt. Every path and pass, every glen and mountain ridge has been trodden and mapped by previous adventurers, and nothing remains for the dissecter now, but to follow with his knife the plain directions of others. But the directions given by our guides are not all equally plain, nor are their charts equally correct or intelligible. These plates, however, will impress all who are familiar with human anatomy, that Mr. Maclise is well acquainted with the topography of every thing which he describes, and that he draws from personal observations, made on the spot, and not from the descriptions given by others.

But the plates do not constitute the sole excellence of the volume before us. The text, explanatory of the plates, and the surgical observations are clear and practical, and form, altogether, a most valuable treatise on surgical anatomy.

We do not hesitate to recommend this book as one of the best and cheapest surgical works ever published. F. H. H.

